# What is the benefit of a high-intensive exercise program on health-related quality of life and depression after stroke? A randomized controlled trial

**DOI:** 10.3109/14038196.2010.488272

**Published:** 2010-06-14

**Authors:** Eva Holmgren, Gunilla Gosman-Hedström, Britta Lindström, Per Wester

**Affiliations:** 1 Department of Community Medicine and Rehabilitation, Section of Physiotherapy and the, Ume å University, Umeå; 2 Department of Public Health and Clinical Medicine, Umeå Stroke Center, Ume å University, Umeå; 3 Vårdalinstitutet, the Swedish Institute for Health Sciences, University of Gothenburg, Gothenburg, Sweden; 4 Department of Clinical Neuroscience and Rehabilitation, Institute of Neuroscience and Physiology, The Sahlgrenska Academy, University of Gothenburg, Gothenburg, Sweden

**Keywords:** Accidental falls, cerebrovascular disorders, depression, exercise, quality of life, rehabilitation

## Abstract

The aim of the study was to evaluate the impact of a high-intensive exercise program containing high-intensive functional exercises implemented to real-life situations together with group discussions on falls and security aspects in stroke subjects with risk of falls. This was a pre-specified secondary outcome for this study. For evaluation, Short Form-36 (SF-36) health-related quality of life (HRQoL) and the Geriatric Depression Scale-15 (GDS-15) were used. This was a single-center, single-blinded, randomized, controlled trial. Consecutive >55 years old stroke patients with risk of falls at 3–6 months after first or recurrent stroke were randomized to the intervention group (IG, *n* = 15) or to the control group (CG, *n* = 19) who received group discussion with focus on hidden dysfunctions but no physical fitness training. The 5-week high-intensive exercise program was related to an improvement in the CG in the SF-36 Mental Component Scale and the Mental Health subscale at 3 months follow-up compared with baseline values while no improvement was seen in the IG at this time. For the SF-36 Physical Component Scale, there was an improvement in the whole study group at 3 and 6 months follow-up compared with baseline values without any significant changes between the IG and CG. The GDS-15 was unchanged throughout the follow-up period for both groups. Based on these data, it is concluded that high-intensive functional exercises implemented in real-life situations should also include education on hidden dysfunctions after stroke instead of solely focus on falls and safety aspects to have a favorable impact on HRQoL.

## Background

A stroke is a life-breaking event that most often hits with no warning, giving no time for preparation for a new way of living the everyday life. It has been established that stroke has direct and/or secondary effects on the major aspects of health (physical, physiological and social) (1,2).

Moderate and high levels of physical activity have been showed to be associated with reduced risk of ischemic and hemorrhagic stroke (3). Physical activity has not been proven to enhance the quality of life (QoL) after stroke according to the Cochrane review on physical fitness training for stroke patients (4). In the same review, the authors state that too few studies have been done to explore any effects of physical fitness training on mood (4). Improvement in activities of daily living (ADL) is known to enhance long-term health-related QoL (HRQoL) (5). According to the most recent Cochrane review on depression and exercise, physical activity seems to decrease depressive symptoms in people with a diagnosis of depression (6).

Health and QoL, the two components of HRQoL, have been linked together in many definitions but there is no universal definition for either QoL or HRQoL. The WHO definition of health is: “Health is a state not merely the absence of disease or infirmity” (7). This is a well-cited and useful definition and it is applicable for everyone. There are many different definitions of HRQoL. According to the Swedish manual of the Short Form-36 (SF-36), HRQoL means a pragmatic delimitation and concerns mainly function and well-being during illness and treatment (8). According to the WHO, depression is a common mental disorder that presents with depressed mood, loss of interest or pleasure, feelings of guilt or low self-worth, disturbed sleep or appetite, low energy, and poor concentration (9). These problems can become chronic or recurrent and lead to substantial impairments in an individual's ability to take care of his or her everyday responsibilities (10). Depression also adversely affect adherence to treatment for other diseases and is among the leading causes of disability worldwide (10). Post-stroke depression is a common complication and has a prevalence of up to 33% (11,12). Data from Riks-Stroke, the Swedish Stroke Register, shows that women are more likely to be depressed than men at 3 months after stroke onset (13). Jorgensen et al. (14) showed in their study from 2002 that depressive symptoms predict falls after stroke. Depression is a common and important complication after stroke but is unclear (11).

A recently published review by Blake et al. concludes (15) that exercise intervention exerts a clinically relevant effect on depressive symptoms in older people (15). However, they do point out a gap of consistent results for the medium-term (3–12 months) effect of exercise intervention on depression or depressive symptoms (15). A task-specific intervention designed to improve gait speed has been showed to have secondary ben efits by positively impacting depression, mobility and social participation for people post-stroke (16).

One could expect that many dimensions of a post-stroke individual could be affected, not only the physical part. The psychological aspects are important for the entire rehabilitation process and for the individuals’ outcome of the same process. It is therefore important to preserve both functioning and well-being of people with stroke. Thus, there is a paucity of data from exercise intervention studies, on whether exercise/rehabilitation programs also have an effect on HRQoL and depression in elderly persons post-stroke.

The accompanying paper with the first report from this intervention study evaluated the impact of a high-intensive exercise program after stroke on function and activity performance (17). The program could be beneficiary in ADL 6 months after the intervention ended and for the Falls Efficacy Scale International (FES-I) directly after and 3 months post-intervention for the intervention group (IG) compared with the control group (CG) (*p*<0.05).

The purpose of the present study was to evaluate the impact of a 5-week high-intensive exercise program (in the IG) or group discussion about hidden dysfunctions after stroke and how to cope with these difficulties (in the CG) on the HRQoL and on the presence of depressive symptoms among individuals with stroke and risk of falls.

## Methods

### Study design

This randomized controlled intervention trial, designed for individuals with stroke and risk of falls, is described in the accompanying paper and is registered at www.clinicaltrials.gov as NCT00377689.

### Subjects

Inclusion criteria were: current stroke, age >55, fall risk assessed through clinical observations by an experienced PT, the ability to walk 10 m with or without a walking device, and the ability to understand and comply with instructions in Swedish. Individuals were excluded if they had the ability to walk outdoors independently (i.e. without assistance or walking device), severe aphasia, severe vision or hearing impairment, any medical condition that a physician determined was inconsistent with study participation, and living too far away (>100 km) from the training facilities.

### Screening and randomization processes

**Figure 1:**
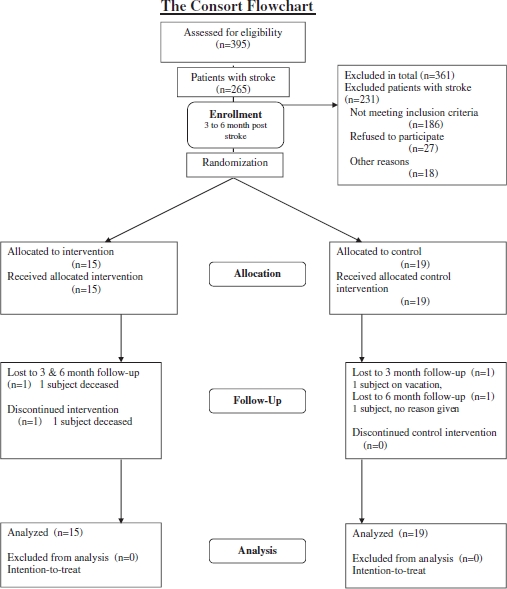
Screening process, from stroke onset to fi nal inclusion in the study.

Inclusion to the study was done 3–6 months after stroke onset. To identify all potentially eligible individuals with stroke, 391 individuals were consecutively screened during inpatient rehabilitation at the Umeå Stroke Unit (Figure 1). After this initial screening, all eligible participants were contacted. Those still eligible for participation in the study a more thorough assessment was performed at the outpatient Clinical Research Center at the Umeå University Hospital. The final judgment of participation in the study was made based on the inclusion/exclusion criteria during this assessment that was considered as the individual's baseline assessment. Figure 1 describes the screening/inclusion process.

The patient enrolment during the inclusion/exclusion assessments was followed by the randomization procedure. The two main investigators (EH and PW) were responsible for the randomization into the IG or CG. This was conducted with a minimization software program, MiniM (18) to avoid imbalances at baseline between the two groups. Two variables were taken into account: cognition, using the Mini Mental State Examination (MMSE <24/>25) (19), and fall risk, using the Fall Risk Index (value <1/>2) (20).

Information about the study was communicated orally and via written materials to all potentially eligible individuals during inpatient rehabilitation, screening phone calls and at the time of inclusion in the study. All individuals provided informed written consent for study participation at the baseline assessments. The study protocol was approved by the local Ethics Committee for Human Research at Umeå University (Dnr 04-022).

### Assessments

A protocol was completed at baseline concerning personal characteristics, living situation, medical history, medication and history of falls. All assessments, baseline, immediately after intervention and 3-month follow-up, were performed at the outpatient Clinical Research Center. The 6-month follow-up assessment was conducted by telephone interview using those instruments with the possibility to administer through telephone, e.g. the HRQoL questionnaire (SF-36) and the Geriatric Depression Scale-15 (GDS-15). A questionnaire was used to evaluate compliance with the home exercise program for the IG. The CG received a questionnaire for evaluation of the educational sessions. Both questionnaires included space for personal comments and evaluation of the intervention program by the participants, and were delivered to the assessment personnel at the 3-month follow-up.

### Blinding

The nurses and physiotherapist (PT) who performed the clinical test assessments were blinded to group allocation. The participants were instructed not to reveal anything from their 5 weeks in the study at the different assessment times. If the staff had any suspicion as to which group the participant belonged to they were told to fill out an incidence form. All participants were blinded as for the content of the two different groups before randomization. They only knew that the two groups met different number of days per week. At the time of the study, participants were open to their own group content but not the other group.

### Intervention

Subjects in each group participated in a 5-week intervention program at a clinic. For the IG, the program consisted of seven sessions a week divided over 3 days with individualized group training, supervised by a PT; the focus was on physical activity and functional performance. They also received one session a week for 1 h with educational group discussions about fall risk and security aspects, led by a PT and an Occupational Therapist (OT). The control subjects program consisted of one session a week for 1 h each during the 5-week period. The session was an educational group discussion session led by one OT. The discussions were about hidden dysfunctions after stroke and how to cope with these difficulties. The different themes discussed were chosen on the basis of being relevant to the individual's situation and interesting for them to participate in. The themes included communication difficulties, fatigue, depressive symptoms, mood swings, personality changes and dysphagia. There was, however, no special focus on the risks of falling in these discussions. The intervention program for the IG is described in somewhat more detail in the accompanying paper (17).

### Outcomes

The outcome measures in this study were HRQoL as measured by the SF-36 and symptoms of depression as measured by the GDS-15. The instruments used in this study have been tested for validity and reliability in populations similar to the population in this study (21-24).

HRQoL was assessed with the SF-36, which is a generic instrument measuring self-reported physical and psychological aspects of health (21,22,25). The SF-36 includes eight subscales: Physical Functioning (PF), Role Functioning-physical (RP), Bodily Pain (BP), General Health (GH),Vitality (VT), Social Functioning (SF), Role Functioning-emotional (RE) and Mental Health (MH). The total score in each sub-scale is 100, which indicates a higher degree of perceived health. Besides the eight different scales, there are two dimensions, a Physical dimension (PCS) and a Mental dimension (MCS). The dimensions are calculated by weighing the different load of the eight scales in to these dimensions. These eight subscales are considered to be universal and to represent basal human function and well-being (8). A difference of five points is considered to be of a clinical significance in SF-36 (8,23). The results for the norm population of Sweden in the eight subscales of SF-36 are shown for the age group 75 +, since the mean age for this entire study is 78. Regarding the two dimensions, PCS and MCS, the norm population compared is in another age group, 75–79, since there is no norm figure for the group 75+ consolidated, only 65+ (26).

Symptoms of depression were assessed with the GDS-15, which is a basic screening measure for depression in older adults (24). The GDS-15 screens for depression using 15 questions with a yes/no answer alternative. Depending on age, education and complaints, the score 0–4 indicates normal (no depression), 5–8 mild depression, 9–11 indicates moderate depression and 12–15 a severe depression.

### Statistical analyses

Power (80%) was calculated on the Berg Balance Scale (BBS) (27,28) for the original study (17), which was the primary outcome measure to determine the sample size needed. The estimation was set to detect a significant difference (*p* = 0.05, two-tailed test) of >5 points in BBS. All analyses were performed according to the intention-to-treat principle (29). Descriptive statistics are presented in frequency as means ± SD in Table I. Groups were compared at baseline using the chi-squared or independent samples *t*-test. For chi-square test, either Pearson chi-square was used or, in applicable cases, Fisher's exact test. The participants’ data were used for analysis for as long as they participated in the study. Generalized estimating equations with repeated measure statistics were used to analyze the data over time and taken into consideration the fact that each individual had multiple assessments (Table II). All data analyses were performed using the SPSS software package, version 17.0.

**Table I:** Baseline characteristics of the participants.

	Intervention group, *n* = 15	Control group, *n* = 19
Sex (M/F)	9/6	12/7
Age	77.7 ± 7.6	79.2 ± 7.5
mRS^a^	2.1 ± 0.6	2.1 ± 0.6
Inpatient rehabilitation, days at stroke unit	12.5 ± 5.0	10.9 ± 5.3
Days from stroke onset to study start	139.7 ± 37.3	126.8 ± 28.2
Diagnosis of depression	3	2
Use of medication, SSRI or other anti-depressants	6	4
Use of sleeping pills	4	6
Home-help service	5	9
MMSE^b^	26.3 ± 3.5	25.5 ± 4.4
Fall risk index (19,37)		
No	14	16
Low	1	0
Medium	0	3
High	0	0

Results are presented as proportion or mean ± SD.

a mRS, modified Rankin Scale

b MMSE, Mini Mental State Examination. SSRI, Selective Serotonin Reuptake Inhibitors.

**Table II:** Repeated measure analyses for all outcome measurements at all follow-up assessments; SF-36 and GDS-15.

		Baseline				Post-Intervention				3 months post-intervention				6 months post-intervention			
					
		Norms for the Swedish population aged 75+	Total study population, *n*=34	IG, *n*=15	CG, *n*=19	CI	Total study population, *n*=33	IG, *n*=14	CG, *n*=19	CI	Total study population, *n*=31	IG, *n*=13	CG, *n*=18	CI	Total study population, *n*=31	IG, *n*=13	CG, *n*=18
SF-36																	
PCS	40.1±12.7	30.8±9.6	30.9±8.3	30.8±10.7	−6.9 to 6.8	32.8±11.3	32.2±10.6	33.2±12.0	−7.2 to 9.2	35.3±13.3	35.5±14.7	35.2±12.7	−10.4 to 9.8	35.3±12.8	35.3±13.3	35.4±12.9	−9.7 to 9.7
MCS	49.0±11.4	53.2±9.4	53.6±10.0	52.8±9.2	−7.5 to 5.9	54.6±10.0	54.4±10.3	54.8±10.0	−6.9 to 7.7	53.3±10.1	48.7±12.7	56.7±6.2	0.9 to 15.0	53.3±12.0	50.4±15.0	55.4±9.3	−3.9 to 13.9
SF-36																	
PF	59.0±30.1	45.8±20.8	45.7±21.7	45.8±20.6	−14.8 to 14.9	48.5±24.6	52.1±22.2	45.8±26.6	−24.2 to 11.5	52.1±26.1	56.5±25.5	48.9±26.8	−27.2 to 11.9	48.7±23.6	51.5±18.6	46.7±26.9	−22.6 to 12.9
RP	49.3±43.2	28.7±38.0	25.0±36.6	31.6±39.8	−20.5 to 33.6	33.3±37.9	21.4±27.5	42.1±42.5	−5.9 to 47.2	41.1±39.0	28.9±39.3	50.0±37.4	−7.3 to 49.6	47.6±41.5	44.2±41.0	50.0±42.9	−25.6 to 37.1
BP	63.2±30.2	64.6±26.6	62.0±19.5	66.6±31.5	−14.3 to 23.5	72.2±28.1	67.9±28.0	75.4±28.5	−12.9 to 27.8	70.7±33.2	66.2±32.4	74.0±34.4	−17.2 to 32.8	74.8±30.5	70.6±31.2	77.8±30.4	−15.7 to 30.1
GH	59.8±24.0	56.3±23.7	57.7±20.1	55.1±26.6	−19.5 to 14.2	56.3±26.8	57.8±28.8	55.3±26.1	−22.1 to 17.1	64.4±25.3	61.7±26.0	66.3±25.4	−14.5 to 23.7	62.0±25.1	60.8±24.1	62.8±26.4	−16.9 to 21.0
VT	54.2±30.0	50.6±18.7	51.0±14.7	50.3±21.8	−14.1 to 12.6	57.1±24.1	59.3±23.3	55.5±25.1	−21.3 to 13.7	53.2±23.5	46.2±19.0	58.3±25.7	−5.0 to 29.4	51.8±23.1	46.7±21.6	55.6±24.0	−8.3 to 26.0
SF	79.1±26.2	79.4±25.5	80.0±26.6	79.0±25.4	−19.3 to 17.2	86.0±23.5	84.8±29.1	86.8±19.3	−15.2 to 19.2	88.3±18.2	83.7±23.6	91.7±12.9	−5.5 to 21.5	90.3±20.3	85.6±25.9	93.8±15.0	−6.9 to 23.3
RE	64.0±41.8	81.4±35.0	73.3±42.2	87.7±27.7	−10.1 to 38.9	79.8±35.3	71.4±38.9	86.0±32.0	−10.7 to 39.7	81.7±35.3	66.7±43.0	92.6±24.4	1.1 to 50.8	83.0±34.3	71.8±40.5	90.7±27.6	−6.0 to 43.9
MH	76.1±23.1	77.2±17.3	82.1±13.6	73.3±19.2	−20.8 to 3.1	81.6±17.7	84.6±12.4	79.4±20.8	−18.0 to 7.6	78.7±18.1	74.5±21.6	81.8±14.9	−6.1 to 20.7	79.0±17.8	81.2±11.9	77.3±21.2	−17.3 to 6.5
GDS-15	n/a	3.0±2.1	2.5±1.7	3.4±2.3	−0.6 to 2.4	4.2±2.7	3.1±2.1	5.0±2.8	−0.3 to 3.6	3.3±2.0	3.2±1.2	3.4±2.5	−1.4 to 1.7	3.4±2.4	3.0±1.5	3.7±2.9	−1.4 to 2.5

Results are presented as mean±SD. GDS-15, Geriatric Depression Scale; SF-36, Short Form 36; IG, intervention group; CG, control group; CI, confi dence interval; PCS, Physical Component Scale; MCS, Mental Component Scale; PF, Physical Functioning; RP, Role Functioning-physical; BP, Bodily Pain; GH, General Health; VT, Vitality; SF, Social Functioning; RE, Role Functioning-emotional; MH, Mental Health.

## Results

The study included 34 participants, 15 subjects in the IG and 19 subjects in the CG. There were no significant differences in the baseline characteristics of the two groups (Table I). There was a success of blinding the group allocation to the clinical test assessment staff.

All but one participant completed the 5-week intervention period. Two participants dropped out during follow-up; the reason for dropout was worsening overall medical condition in both cases. The participants in the IG participated in the home exercise program two or three times per week according to the self-reporting questionnaire.

Significant difference between the two groups were detected in SF-36 MCS and MH subscale at the 3-month follow-up in favor of the CG (*p* = 0.02). There were no differences between the IG and the CG in presence of depressive symptoms as measured by GDS-15.

### SF-36 Physical Component Scale (PCS)

The PCS levels are lower than in the norm population for this age bracket (norm population = 40.1, total study population = 30.8) (Table II). There was no difference in SF-36 PCS at baseline between the IG and the CG. At the 3- and 6-month follow-up, there was partial normalization of the SF-36 PCS for the whole study group (IG + CG) vs values at baseline (*p*< 0.05, Table II). However, there was no significant difference for SF-36 PCS between the IG and CG over time (Table II). For the individual components of the SF-36 PCS, PF, RP, BP and GH, there were no significant differences between the IG and the CG at baseline or over time post-intervention (Table II).

### SF-36 Mental Component Scale (MCS)

There is a significant difference in SF-36 MCS between the IG and CG at 3 months follow-up in favor for the CG (*p* = 0.02, Table II). The subscale MH also showed a similar significant difference at the 3-month follow-up (*p* = 0.02). The results for the remaining individual components of the SF-36 MCS, VT, SF and RE were without significant differences between IG and CG at baseline and over time after intervention (Table II).

There were no difference in SF-36 MCS at baseline between the IG and the CG. The MCS levels in whole study group (IG+ CG) were higher than in the norm population for this age bracket (53 vs 49 at baseline measurements, Table II). In the whole study group (IG+CG), there was no difference over time during the study period (Table II).

### Geriatric Depression Scale-15 (GDS-15)

There was no statistically significant difference in GDS-15 between the IG and CG at baseline. There was no assured difference over time in the GDS-15 between the IG and CG. All levels of GDS-15 are without signs of depression.

## Discussion

This 5-week high-intensive exercise program did have an impact on HRQoL. The CG had a favorable outcome in the SF-36 MCS and MH subscale at 3 months post-intervention while no such improvement was observed in the IG. This was the only difference achieved between the two groups regarding HRQoL. For the SF-36 PCS, there was an improvement in the whole study group at 3 and 6 months post-intervention compared with baseline values without any significant changes between the IG and the CG. The presence of depressive symptoms was unchanged throughout the follow-up period for both groups.

The SF-36 MCS was higher for the whole study group compared with the norm population at baseline and directly post-intervention. This means that the stroke subjects in this study at this time point were mentally more vital compared with the general unaffected population. This could be an effect that the stroke patients were still in a stage of recovery and after all had survived. It may take longer than 3–6 months after stroke to adapt to the situation of living with the consequences of the stroke. One year after stroke, many persons with even mild stroke still struggle to cope with these consequences, often hidden dysfunctions (30,31). There was a difference in SF-36 MCS and MH at 3-month follow between the IG and CG vs baseline measurements. The magnitude of these changes was 9 points for SF-36 MCS and 16 points for MH. The clinical significance of SF-36 is set at 5 points or more (8), indicating in the present study that a clinically detectable and meaningful change has occurred.

The reason(s) for the difference between the IG and CG in SF-36 MCS and MH at 3 months post-intervention are unclear. Possible factors may include:

- Different content in the intervention program in the IG and the CG. The group discussions in the IG and the CG were equal in numbers and length but they had a completely different focus. The discussions in the IG concentrated solely on fall risk and security aspects, while the discussions in the CG contained group discussion on hidden dysfunctions after stroke as their complete intervention. The vast majority of the subjects in the CG reported in a questionnaire that they perceived the group discussions as interesting and meaningful with good and open-minded atmosphere within the group.
- Disappointment and frustration in the IG that the intervention had ended and thereby that the subjects had lost their intensive connections to the other participants in the study group as well as to attention loss of the personnel involved in the study. This is supported by the overwhelmingly positive feedback given by the majority of the subjects in the IG to the intervention staff in the study at the end of the 5-week intervention program. Also, many of these subjects expressed a desire to participate repeatedly in high-intensive exercise program rounds.

The main focus of the presently evaluated intervention study was not to investigate the impact on HRQoL and depression but rather to explore the effects on various physical outcomes. The evaluation of HRQoL and depressive symptoms was done in order to see if an “ordinary” exercise program did have an effect on these kinds of outcomes. The study was not designed for effect on these outcomes; therefore these results are in fact confirmatory of this. If effect on these variables, HRQoL and depressive symptoms, are sought after, the study needs to be designed for that as well. It could, however, be considered as a strength in this study, that the different discussion topics in the CG seem to be of importance for HRQoL. For future interventions programs, this knowledge is valuable and needs to be added to the discussion sessions in order to have a possible effect on these outcomes. It is known that the different invisible symptoms such as different cognitive problems are very common, but not often primary will be taken into account during the rehabilitation period. In persons 55 years and younger, cognitive problems are frequently perceived (32). This may be important to absorb and in the design of future programs add these topics to the theory sessions to demonstrate effects on HRQoL.

Regarding depressive symptoms, there was no significant difference over time in the whole study group or between the IG and the CG. Five out of 34 subjects (three in the IG and two in the CG) had a previous history of depression whereas 27% in the IG and 32% in the CG were on antidepressant therapy at baseline measurements. This shows that the proportion of subjects in the present study with treated depression is in the range of previously published data on post-stroke depression (12). However, the average value of GDS-15 was 3.0 in the whole study group at baseline measurements (2.5 in the IG and 3.4 in the CG), indicating that depressive symptoms were not common. This suggests that many of the subjects in our study had a positive effect of their anti-depressive medication. Since the intervention study could have been considered demanding to the post-stroke individuals, both in time as well as physical and mental effort, it is likely that many of these individuals with more pronounced depressive symptoms declined the offer to participate in the study.

The SF-36 has been tested for validity and reliability in populations similar to the population in this study (8,21,23,24). However, there is a debate on some of its subscales, indicating imprecision and confounding when making summed scores (33). The results from the subscales of importance in this study nevertheless support the generation of summed scores from PF, RP, BP, VT, RE and MH (2). The Stroke Impact Scale (SIS) is another possible instrument that may be suitable for a post-stroke sample (34). The SIS instrument is disease-specific and results in values on physical symptoms, function, activity, ability/trouble in daily life, the impact of the disease, satisfaction and patient satisfaction (34). Both SIS and SF-36 are generic instruments, but in contrast to the SIS, the SF-36 is not limited to be disease-specific. Generic instrument in general, evaluates the patient's general health and QoL. An advantage of using SF-36 is that it could be used to compare the results with other studies of other diseases using the SF-36. In our study population, the mean age was 79 years and the vast majority of the subjects had a high co-morbidity with illnesses, such as cardiovascular and other diseases. It might therefore be difficult to ensure the disease-specific origin of the symptoms. Therefore, a generic instrument rather than a disease-specific instrument has been considered as best relevant to use in this study.

As has been discussed in the accompanying report, this sample was considered representative for individuals with post-stroke symptoms of fall risk and the length of the intervention program is realistic for implementation in clinic (17). Fatigue as a common complication after stroke was also given as the main reason not to participate by those who declined to participate in this study. Of those eligible for inclusion in the study but were not included in the study, some lived too far away from the rehabilitation facilities. A few others did not have the time to spend in the intervention program, while some others died during the time between inpatient care and study start. With these facts, it might be that our included study group is a bit more active than the average post-stroke individuals with risk of falls. However, many participants in the study group expressed a pronounced fatigue.

In the present study, the mean result of the SF-36 subscale VT (which is defined as either feeling tired all the time or having lots of energy all of the time (8)) indicates that this proportion was higher than in the norm population. VT has been strongly associated with global ratings of health satisfaction and QoL (8).

There are several limitations in this study (17). The size of the study population is small. The power calculation was based on the BBS and estimated minimum size of 34 subjects. To reach a high precision in SF-36, there is a call for more than 200 subjects (8). This suggests that there is a possibility for a Type-2 statistical error in other subscales of SF-36 in the present study, i.e. there may be a difference that is non-detectable because of our small sample size.

We consider it a strength to try to evaluate HRQoL and depression, and not only functional and activity measures, even though the intervention study is focused on these outcomes. It is established that both physical and psychosocial well-being is greatly affected in stroke survivors (1). Many rehabilitation programs are focused on the physical outcome of the program. There is a need for evaluating rehabilitation programs in terms of HRQoL and the presence of depressive symptoms. It could also be concluded, to achieve an effect on depressive symptoms, that the program should have included exercise that focused on higher level of aerobic training (33,36).

Antidepressant drugs may be useful in treating depression after stroke, but can also cause side-effects, especially such as seizures, falls and delirium (37). Adding these side-effects to an already decreased functional ability may be problematic. Therefore, an alternative method as physical activity may be a useful way to ameliorate depressive symptoms. Ideally, in order to evaluate the effect of physical fitness training on depression after stroke, the participating stroke individuals should be medication-free regarding anti-depressants. When designing the structured intervention program, the focus of the study was on functional rehabilitation, with an attention time of 30 h during the 5-week intervention program for the IG and 5 h for the CG and actually not to enhance the HRQoL. The psychosocial part was incorporated in the 1 h/week of education received by the individuals in both groups.

## Conclusions

Our 5-week high-intensive exercise program was related to deterioration in the IG in SF-36 MCS and the MH subscale at 3 months post-intervention compared with baseline values while the CG improved at this time. For the SF-36 PCS, there was an improvement in the whole study group at 3 and 6 months post-intervention compared with baseline values without any significant difference between the IG and the CG. The presence of depressive symptoms was unchanged throughout the follow-up period for both groups. Based on these data, it is concluded that a modified version of a high-intensive exercise program to be tested in the future should not entirely focus on falls and safety aspects, but should also include themes on hidden dysfunctions after stroke in order to have a favorable impact on HRQoL.

## References

[1] Carod-Artal FJ, Egido JA (2009). Quality of life after stroke: The importance of a good recovery. Cerebrovasc Dis.

[2] Hobart JC, Williams LS, Moran K, Thompson AJ (2002). Quality of life measurement after stroke: Uses and abuses of the SF-36. Stroke.

[3] Lee CD, Folsom AR, Blair SN (2003). Physical activity and stroke risk: A meta-analysis. Stroke.

[4] Saunders DH, Greig CA, Mead GE, Young A (2009). Physical fitness training for stroke patients. Cochrane Database Syst Rev.

[5] Indredavik B, Bakke F, Slordahl SA, Rokseth R, Haheim LL (1998). Stroke unit treatment improves long-term quality of life: A randomized controlled trial. Stroke.

[6] Mead GE, Morley W, Campbell P, Greig CA, McMurdo M, Lawlor DA (2008). Exercise for depression. Cochrane Database Syst Rev.

[7] WHO (1946). WHO definition of Health.

[8] Sullivan M, Karlsson J, Taft C (2002). Swedish manual and interpretation guide.

[10] WHO (2008). Scaling up care for mental, neurological, and substance use disorders. Programme MHGA.

[11] Hackett ML, Anderson CS, House A, Halteh C (2008). Interventions for preventing depression after stroke. Cochrane Data base Syst Rev.

[12] Hackett ML, Yapa C, Parag V, Anderson CS (2005). Frequency of depression after stroke: A systematic review of observational studies. Stroke.

[13] Register TSS (2007). RIKS-Stroke, annual report 2007.

[14] Jørgensen L, Engstad T, Jacobsen B (2002). Higher incidence of falls in long-term stroke survivors than in population controls: Depressive symptoms predict falls after stroke. Stroke.

[15] Blake H MP, Malik S, Thomas S (2009). How effective are physical activity interventions for alleviating depressive symptoms in older people? A systematic review. Clin Rehabil.

[16] Smith PS, Thompson M (2008). Treadmill training post stroke: Are there any secondary benefits? A pilot study. Clin Rehabil.

[17] Holmgren E, Gosman-Hedström G, Lindström B, Nyberg L, Wester P (2010). What is the benefit of a high intensive exercise program after stroke?—A randomized controlled trial. Adv Physiother.

[18] Evans S, Royston P, Day S (2004). Minim: Allocation by minimisation in clinical trials. http://www-users.york.ac.uk/-mb55/guide/minim.htm.

[19] Folstein MF, Folstein SE, McHugh PR (1975). “Mini-mental state". A practical method for grading the cognitive state of patients for the clinician. J Psychiatr Res.

[20] Olsson E, Lofgren B, Gustafson Y, Nyberg L (2005). Validation of a fall risk index in stroke rehabilitation. J Stroke Cerebrovasc Dis.

[21] Anderson C, Laubscher S, Burns R (1996). Validation of the Short Form 36 (SF-36) health survey questionnaire among stroke patients. Stroke.

[22] Sullivan M, Karlsson J, Ware JE (1995). The Swedish SF-36 Health Survey—I. Evaluation of data quality, scaling assumptions, reliability and construct validity across general populations in Sweden. Soc Sci Med.

[23] Ware JE, Sherbourne CD (1992). The MOS 36-item short-form health survey (SF-36). I. Conceptual framework and item selection. Med Care.

[24] Yesavage JA, Brink TL, Rose TL, Lum O, Huang V, Adey M (1982). Development and validation of a geriatric depression screening scale: A preliminary report. J Psychiatr Res.

[25] Ware JE, Snow KK, Kosinski M, Gandek B (1993). SF-36 Health Survey: Manual and interpretation guide.

[26] Sullivan M, Taft C (2002). SF-36 Hälsoenkät Swedish manual and interpretation guide.

[27] Berg K, Wood-Dauphinee S, Williams JI (1995). The Balance Scale: Reliability assessment with elderly residents and patients with an acute stroke. Scand J Rehabil Med.

[28] Berg KO, Wood-Dauphinee SL, Williams JI, Maki B (1992). Measuring balance in the elderly: Validation of an instrument. Can J Public Health.

[29] Altman DG (1991). Practical Statistics for medical research.

[30] Carlsson GE, Moller A, Blomstrand C (2004). A qualitative study of the consequences of ‘hidden dysfunctions’ one year after a mild stroke in persons <75 years. Disabil Rehabil.

[31] Carlsson GE, Moller A, Blomstrand C (2009). Managing an everyday life of uncertainty — A qualitative study of coping in persons with mild stroke. Disabil Rehabil.

[32] Roding J, Glader EL, Malm J, Eriksson M, Lindstrom B (2009). Perceived impaired physical and cognitive functions after stroke in men and women between 18 and 55 years of age — A national survey. Disabil Rehabil.

[33] Svensson E (2001). Guidelines to statistical evaluation of data from rating scales and questionnaires. J Rehabil Med.

[34] Duncan PW, Wallace D, Lai SM, Johnson D, Embretson S, Laster LJ (1999). The stroke impact scale version 2.0. Evaluation of reliability, validity, and sensitivity to change. Stroke.

[35] Oeland AM, Laessoe U, Olesen AV, Munk-Jorgensen P (2010). Impact of exercise on patients with depression and anxiety. Nord J Psychiatry.

[36] Rimmer JH, Wang E (2005). Aerobic exercise training in stroke survivors. Top Stroke Rehabil.

[37] Hackett ML, Anderson CS, House A, Xia J (2008). Interventions for treating depression after stroke. Cochrane Database Syst Rev.

[38] Nyberg L, Gustafson Y (1997). Fall prediction index for patients in stroke rehabilitation. Stroke.

